# Prominent T-Cell Responses against the Acetylcholine Receptor *ε* Subunit in Myasthenia Gravis

**DOI:** 10.1155/2019/1969068

**Published:** 2019-03-03

**Authors:** Oliver Neuhaus, Karl-Heinz Wiesmüller, Hans-Peter Hartung, Heinz Wiendl

**Affiliations:** ^1^Department of Neurology, Medical University of Graz, Graz, Austria; ^2^Department of Neurology, SRH Kliniken Landkreis Sigmaringen GmbH, Sigmaringen, Germany; ^3^EMC Microcollections GmbH, Tübingen, Germany; ^4^Department of Neurology, University of Düsseldorf, Düsseldorf, Germany; ^5^Department of Neurology, University of Tübingen, Tübingen, Germany; ^6^Department of Neurology, University of Münster, Münster, Germany

## Abstract

The human acetylcholine receptor (AChR) is well characterized as the target antigen in myasthenia gravis (MG). Pathogenic antibody responses against the AChR alpha-chain have been investigated extensively and are of diagnostic and prognostic value. However, less is known on the pathogenetic relevance of T-cell responses against epitopes of the different AChR chains (alpha, epsilon, gamma). Using an enzyme-linked immunospot (ELISPOT) assay we measured T-cell responses against recombinant fragments and synthetic peptides of the *α* and the *ε* subunits of the human AChR in MG patients (n=15) and in healthy donors (HD; n=9). In MG, highest T-cell responses were noted against recombinantly expressed Epsilon 1-221. Among the synthetic peptides Epsilon 201-215 showed the most prominent T-cell response and represented the peptide with the most remarkable difference between MG and HD. Taken together, prominent T-cell responses against the *ε* subunit of the human AChR indicate an important role in the pathogenesis of MG.

## 1. Introduction

The human nicotinic acetylcholine receptor (AChR) composed of five subunits (2 *α*, 1 *β*, 1 *δ*, and either 1 *γ* or 1 *ε* subunit) is well characterized as the target antigen in myasthenia gravis (MG) [1]. The *γ* subunit of the fetal receptor is replaced by an *ε* subunit in adult muscle; both subunits share about 53% homology at the amino acid level [2]. Pathogenic antibodies are predominantly directed against the *α* subunit of the AChR. Both antibody responses as well as B-lymphocyte activity have been investigated extensively in MG and are of great diagnostic and prognostic value.

Immunoglobulin G (IgG) autoantibody production is T helper cell-dependent. Although MG is considered a prototypic paradigm for an antibody-driven autoimmune disorder, the pathogenetic importance of T-helper cells is well appreciated. Several studies have been performed comparing T-cell responses involving the *α* subunit versus the developmentally regulated *ε* subunit using recombinant fragments and purified polypeptides of the human AChR [2–8]. The *ε* subunit is of particular interest as its expression in the adult muscle differs from the fetal *γ* subunit, a fact that may contribute to the escape of clonal deletion and the development of autoreactive T lymphocytes in MG, especially in the myasthenic thymus [9]. In accordance with this hypothesis, two reports describe that, in comparison to healthy subjects, only MG patients responded to synthetic peptides of the *ε* subunit by T-cell proliferation [2, 10]. Consistently, in MG patients with thymomas the *ε* subunit is preferentially expressed [11].

We used an enzyme-linked immunospot (ELISPOT) assay to determine T-cell responses against recombinant fragments and synthetic peptides of the human AChR. In accordance with previous observations we found prominent T-cell responses against the *ε* subunit while no significant differences were notable against alpha subunit epitopes.

## 2. Patients and Methods

### 2.1. Patients and Controls

Peripheral blood lymphocytes (PBL) were obtained with informed consent from patients with generalized or ocular MG (n=15) or healthy donors (HD, n=9). PBL were isolated by density centrifugation and were either frozen immediately and thawed for analysis or used directly.

### 2.2. Synthesis of Recombinant Fragments

Human *α* and *ε* subunit polypeptides were synthesized by PCR on cDNA prepared from total RNA of human calf muscle as described elsewhere [12]. Recombinant protein fragments were kindly provided by Wolfgang Wienhold and Arthur Melms [13]. Fragments were expressed in* E. coli* and purified by SDS/PAGE with a standard protocol [5, 12]. Alpha 1-103 and Alpha 1-209 are fragments of the extracellular domain, Alpha 327-298 of the intracellular domain of the *α* subunit [1]. Epsilon 1-221 is a fragment of the extracellular domain of the *ε* subunit.

### 2.3. Synthesis of Peptides

Peptides were synthesized by solid-phase Fmoc-chemistry on an automated peptide synthesizer for multiple peptide synthesis as described previously [14]. Epsilon 116-130, IDGQFGVAYDANVLV, is an HLA-DR3-binding peptide. Epsilon 201-215, ENGEWAIDFCPGVIR, contains a dominant epitope restricted by HLA-DR52a [7]. Epsilon 236-250, IRRKPLFYVINIIVP, contains a dominant T-cell epitope that is not HLA-DR3-restricted. As a specificity control peptide, we used the class II-associated invariant chain peptides, CLIP 97-120, LPKPPKPVSKMRMATPLLMQALPM, and CLIP 105-117, SKMRMATPLLMQA. The tetanus toxoid peptide TT 1272-1284 is a promiscuitive HLA-DR3/DR52a binder.

### 2.4. ELISPOT Assay

We measured frequencies of interferon (IFN)-*γ*-secreting T-cells using an ELISPOT (enzyme-linked immunospot) assay as described previously [15]. Microtiter filter plates (Millipore) were coated overnight with an anti-human IFN-*γ* monoclonal antibody (mAb) (clone 1-D1K; 10 *μ*g/ml, Mabtech, Sweden). After washing and blocking the plates with culture medium (RPMI 1640 supplemented with 5% fetal bovine serum and antibiotics, all from Gibco), fresh or freshly thawed PBL from MG patients and HD were incubated for 20 h in duplicate in the presence or absence of human AChR antigens or controls (1 *μ*g/ml). Concanavalin A (5 *μ*g/ml; Sigma) was used as positive control. Using biotinylated anti-human IFN-*γ* mAb (clone 7-B61; Mabtech), streptavidin-alkaline phosphatase (Mabtech), and BCIP/NBT as substrate (Sigma), antigen-specific IFN-*γ* secreting T lymphocytes were visualized and counted on a dissecting microscope. Results are calculated and assigned as the numbers of IFN-*γ*-secreting events among 10^6^ PBL minus the corresponding numbers of events per 10^6^ PBL without antigen.

### 2.5. Statistical Analysis

Student's* t*-test was performed for statistical analysis. A p value of < 0.05 was accepted to be significant.

## 3. Results and Discussion

**Figure 1:**
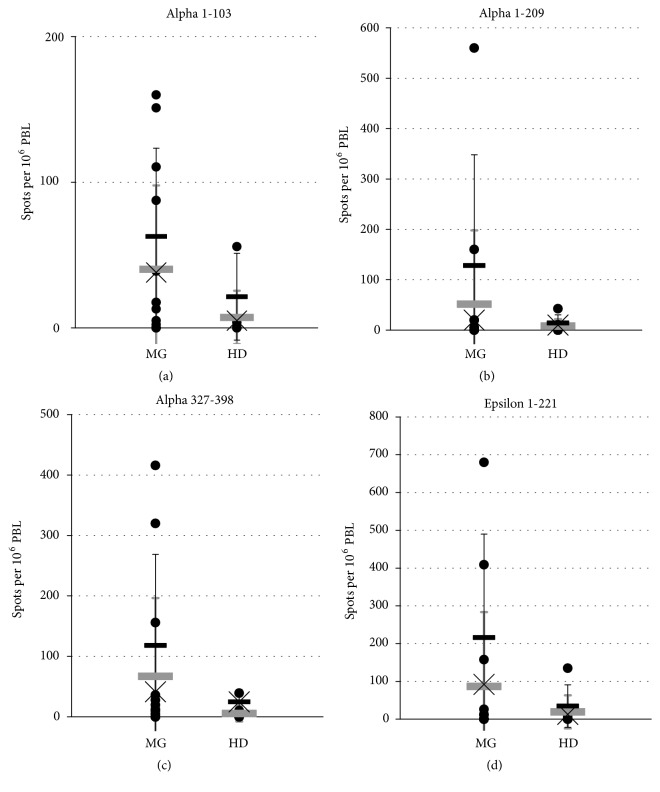
Frequency of IFN-*γ*-secreting events per 10^6^ PBL in single donors. ELISPOT assay was performed as described in the text. Results are assigned as the numbers of IFN-*γ*-secreting events among 10^6^ PBL minus the corresponding numbers of events per 10^6^ PBL without antigen. Negative results (spot number without antigen exceeding spot number with antigen) were defined zero. Grey bars, mean response ± SD; black bars, mean positive response ± SD; crosses, median positive response; MG, myasthenia gravis patients; HD, healthy donors. Note the high standard deviations due to heterogeneous responses of single individuals (see Table 1). Note the different Y axis scales using four different recombinant fragments. (a) fragment Alpha 1-103; (b) Alpha 1-209; (c) Alpha 327-398; (d) Epsilon 1-221.

**Figure 2:**
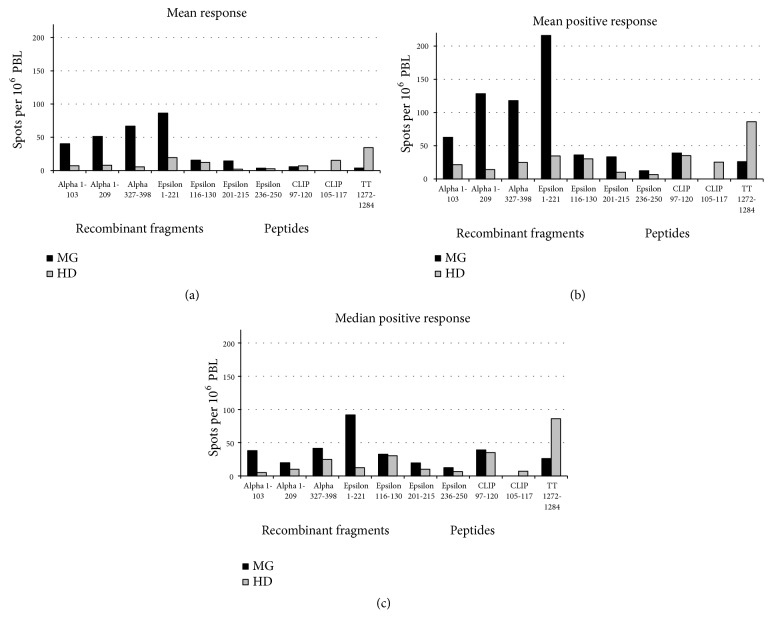
(a) Mean response, (b) mean positive response, and (c) median response of the numbers of IFN-*γ*-secreting events among 10^6^ PBL minus the corresponding numbers of events per 10^6^ PBL without antigen. Negative results (spot number without antigen exceeding spot number with antigen) were defined zero. MG, myasthenia gravis patients; HD, healthy donors.

The T-cell responses were heterogeneous throughout MG patients. While single individuals did not show any detectable IFN-*γ* secreting T-cells after stimulation with AChR fragments (Table 1), others exhibited marked responses (Figures 1 and 2). The responses did not correlate with the AChR-antibody status. For example, patient MG-8 was AChR-antibody negative but exhibited a T-cell response to the AChR protein fragments. Accordingly, some HD presumably AChR-antibody negative gave positive T-cell responses. Analyzing the mean responses of all donors, the mean of detectable (positive) responses only, or the median positive responses only, recombinant fragments of both the *α* and the *ε* subunit induced a higher T-cell response in MG than in HD (Table 1). However, statistical analysis could only determine a trend and not statistical significance.

The fragment Epsilon 1-221 showed the highest response. Synthetic peptides of the *ε* subunit induced a lower response. The most remarkable difference between MG and HD was observed with Epsilon 201-215 containing a dominant T-cell epitope (Table 1) [7]. Consistent with this finding, Ragheb and colleagues have demonstrated proliferative T-cell responses upon stimulation with synthetic peptides of the *ε* subunit in up to 15% of MG patients including Epsilon 194-209 [2].

Correlations between AChR-specific T-cell responses and paraclinical data (sex, age, or anti-AChR antibody serum titer) were not observed (see Table 1). In an animal model of MG, experimental autoimmune myasthenia gravis (EAMG), Gaertner et al. investigated the pathogenicity of T-cell determinants of the *ε* subunit [8]. Although IFN-*γ* secretion by T-cells reactive to *ε* subunit peptides was observed, these cells failed to induce EAMG upon transfer. Hence, these T lymphocytes were demonstrated to be nonpathogenic. It remains to be determined if this observation reflects a peculiarity of the EAMG model or if nonpathogenic, *ε* subunit-specific T lymphocytes are present in MG patients. If so, it is speculated that they may contribute to the integrity of the neuromuscular junction [8].

We conclude that the IFN-*γ* ELISPOT method may provide a valuable tool to measure AChR-specific T-cell responses in MG. In MG patients who tested positive, T lymphocytes specific for epitopes of the AChR *ε* subunit may be a target for therapeutical intervention in MG.

## Figures and Tables

**Table 1:** 

Group	Donor	Sex (f/m)	Age (years)	AChR-Ab (nmol/l)		Spots per 10^6^ PBL									
						Recombinant fragments				Peptides					
					No	Alpha	Alpha	Alpha	Epsilon	Epsilon	Epsilon	Epsilon	CLIP	CLIP	TT
					antigen	1-103	1-209	327-398	1-221	116-130	201-215	236-250	97-120	105-117	1272-1284
MG	MG-1	f	70	135.6	1	0	0	0	0	n.d.	n.d.	n.d.	n.d.	n.d.	n.d.
	MG-2	f	62	0.5	11	0	0	0	0	n.d.	n.d.	n.d.	n.d.	n.d.	n.d.
	MG-3	m	48	10.2	2	160	560	416	680	n.d.	n.d.	n.d.	n.d.	n.d.	n.d.
	MG-4	m	66	0.0	10	0	0	36	0	n.d.	n.d.	n.d.	n.d.	n.d.	n.d.
	MG-5	m	50	2.2	5	151	160	320	409	n.d.	n.d.	n.d.	n.d.	n.d.	n.d.
	MG-6	m	64	0.4	12	2	0	28	0	n.d.	n.d.	n.d.	n.d.	n.d.	n.d.
	MG-7	f	66	0.0	38	39	0	5	0	n.d.	n.d.	n.d.	n.d.	n.d.	n.d.
	MG-8	m	71	0.0	2	88	20	10	158	n.d.	n.d.	n.d.	n.d.	n.d.	n.d.
	MG-9	m	58	2.8	13	5	5	0	13	0	0	0	0	0	0
	MG-10	f	82	8.1	6	18	5	0	0	0	3	5	0	0	0
	MG-11	f	25	76.9	20	18	0	13	0	0	0	0	0	0	0
	MG-12	f	36	1.7	39	0	0	0	0	0	0	0	0	0	0
	MG-13	f	53	1.5	1	13	20	20	26	33	20	20	39	0	26
	MG-14	f	38	46.0	3	0	0	0	11	43	0	0	0	0	0
	MG-15	f	32	22.2	134	111	0	156	0	33	78	0	0	0	0

Mean response			**54.7 **	**20.5 **	**19.8 **	**40.2 **	**51.3 **	**66.8 **	**86.4 **	**15.5 **	**14.3 **	**3.5 **	**5.6 **	**0.0 **	**3.7 **
SD			**16.4**	**38.5**	**33.9**	**57.5**	**146.5**	**129.7**	**197.0**	**19.6**	**29.0**	**7.3**	**14.7**	**0.0**	**9.8**
Mean positive response						**62.7**	**128.3**	**118.0**	**216.0**	**36.1**	**33.3**	**12.3**	**39.0**	**0.0**	**26.0**
SD						**60.7**	**219.7**	**150.9**	**274.1**	**6.2**	**39.6**	**10.3**			
Median positive response						**38.0**	**19.8**	**41.5**	**91.8**	**32.5**	**19.5**	**12.3**	**39.0**	**0.0**	**26.0**

HD	HD-1	m	47	n.d.	2	0	5	10	13	n.d.	n.d.	n.d.	n.d.	n.d.	n.d.
	HD-2	f	26	n.d.	3	0	10	0	3	n.d.	n.d.	n.d.	n.d.	n.d.	n.d.
	HD-3	f	30	n.d.	1	0	0	0	0	n.d.	n.d.	n.d.	n.d.	n.d.	n.d.
	HD-4	f	32	n.d.	19	56	0	39	0	n.d.	n.d.	n.d.	n.d.	n.d.	n.d.
	HD-5	m	79	n.d.	4	5	3	0	13	0	0	3	0	0	0
	HD-6	f	64	n.d.	49	0	43	0	135	50	10	0	35	0	165
	HD-7	f	53	n.d.	1	4	11	0	11	11	0	11	0	7	7
	HD-8	f	48	n.d.	6	0	0	0	0	0	0	0	0	6	0
	HD-9	m	60	n.d.	95	0	0	0	0	0	0	0	0	63	0

Mean response			**48.8 **		**20.0 **	**7.1 **	**7.9 **	**5.5 **	**19.3 **	**12.1 **	**2.0 **	**2.6**	**7.0 **	**15.2 **	**34.4 **
SD			**17.5**		**32.1**	**18.3**	**13.7**	**13.1**	**43.8**	**21.7**	**4.5**	**4.5**	**15.7**	**26.7**	**73.1**
Mean positive response						**21.4**	**14.1**	**24.8**	**34.7**	**30.3**	**10.0**	**6.5**	**35.0**	**25.3**	**86.0**
SD						**29.8**	**16.2**	**20.3**	**56.2**	**27.9**		**5.7**		**32.2**	**111.7**
Median positive response						**5.0**	**10.0**	**24.8**	**12.5**	**30.3**	**10.0**	**6.5**	**35.0**	**7.0**	**86.0**

ELISPOT assay was performed as described in the text. Results are assigned as the numbers of IFN-gamma-secreting events among 10^6^ PBL minus the corresponding numbers of events per 10^6^ PBL without antigen. Negative results (spot number without antigen exceeding spot number with antigen) were defined zero. MG, myasthenia gravis patients; HD, healthy donors; n.d., not done.

## Data Availability

Data supporting the results of this study can be provided by the corresponding author.
